# Long non-coding RNA HOTAIR promotes glioblastoma cell cycle progression in an EZH2 dependent manner

**DOI:** 10.18632/oncotarget.2681

**Published:** 2014-11-04

**Authors:** Kailiang Zhang, Xiaotian Sun, Xuan Zhou, Lei Han, Luyue Chen, Zhendong Shi, Anling Zhang, Minhua Ye, Qixue Wang, Chaoyong Liu, Jianwei Wei, Yu Ren, Jingxuan Yang, Jianning Zhang, Peiyu Pu, Min Li, Chunsheng Kang

**Affiliations:** ^1^ Department of Neurosurgery, Tianjin Medical University General Hospital; Laboratory of Neuro-Oncology, Tianjin Neurological Institute; Key Laboratory of Post-trauma Neuro-repair and Regeneration in Central Nervous System, Ministry of Education; Tianjin Key Laboratory of Injuries, Variations and Regeneration of Nervous System, Tianjin, China; ^2^ Department of Medicine, Department of Surgery, The University of Oklahoma Health Sciences Center, Oklahoma City, OK, USA; ^3^ Department of Gastroenterology, Changhai Hospital, Second Military Medical University, Shanghai, China; ^4^ The Department of Otorhinolaryngology and Maxillofacial Oncology, Tianjin Medical University Cancer Institute and Hospital; Key Laboratory of Cancer Prevention and Therapy, Tianjin Cancer Institute; National Clinical Research Center of Cancer, Tianjin, China; ^5^ Department of Neurosurgery, Second Affiliated Hospital of Nanchang University, Nanchang, Jiangxi Province, China; ^6^ School of Materials Science and Engineering, Tianjin University, Tianjin, China; ^7^ Tianjin Research Center of Basic Medical Science, Tianjin Medical University, Tianjin, China

**Keywords:** Long non-coding RNA, HOTAIR, GBM, Cell cycle, EZH2

## Abstract

The long non-coding RNA Hox transcript antisense intergenic RNA (HOTAIR) was recently implicated in breast cancer metastasis and is predictive of poor prognosis in colorectal and pancreatic cancers. We recently discovered that HOTAIR is a cell cycle-related lncRNA in human glioma, and its expression is closely associated with glioma staging and poor prognosis. Although lysine specific demethylase 1 (LSD1) and polycomb repressive complex 2 (PRC2) have been demonstrated to be functional targets of HOTAIR, how HOTAIR regulates glioma cell cycle progression remains largely unknown. In this study, we found that EZH2 (predominant PRC2 complex component) inhibition blocked cell cycle progression in glioma cells, consistent with the effects elicited by HOTAIR siRNA. However, the inhibition of LSD1 did not affect cell cycle progression in glioma cells. These results suggest that HOTAIR might regulate cell cycle progression through EZH2. Our intracranial mice model also revealed delayed tumor growth in HOTAIR siRNA- and EZH2 inhibitor-treated groups. Moreover, in HOTAIR knock-down cell lines, the expression of the PRC2-binding domain of HOTAIR (5′ domain) but not of the LSD1-binding domain of HOTAIR (3′ domain) resulted in accelerated cell cycle progression. In conclusion, HOTAIR promotes cell cycle progression in glioma as a result of the binding of its 5′ domain to the PRC2 complex.

## INTRODUCTION

Glioblastoma (GBM) represents the most aggressive and deadly primary brain tumor. The median survival of GBM patients is approximately 12 months from the time of diagnosis [1]. Patients benefit from maximal surgical resection, followed by radiotherapy and chemotherapy [2-3]. However, GBM cannot be fully resected because of its infiltrative growth. It is difficult to find a balance between the extent of resection and neurological morbidity. Even when maximal resection is achieved, tumor recurrence has been detected within the radiation field in most patients [4]. The cell cycle lies at the heart of cancer, and deregulated cell cycle progression can result in uncontrolled cancer cell proliferation. Temozolomide (TMZ) is an alkylating agent that is used in the treatment of GBM. TMZ therapy elicits anti-tumor activity by alkylating/methylating DNA at the N-7 or O-6 positions of guanine residues. As a result, TMZ induces DNA damage and death of tumor cells. Although TMZ has been used to treat newly diagnosed GBM since 2005 as a standard-of-care treatment, the median survival time of all GBM patients after diagnosis remains less than 12 months [5]. These findings indicate that cell cycle progression is complicated in GBM. Thus, understanding the underlying mechanisms that regulate cell cycle progression in GBM might provide significant insight into the enhancement of the therapeutic management of GBM.

Non-coding RNAs (ncRNAs) are functional RNAs that do not encode proteins. NcRNAs include microRNAs (miRNAs), long non-coding RNAs (lncRNAs), tRNAs, snoRNAs, and siRNAs [6-7]. miRNAs have garnered significant attention over the past decade. Increasing studies have demonstrated that miRNAs are involved in the proliferation, invasion, apoptosis and cell cycle progression of cancer cells by binding to their mRNA targets [8-12]. Different from miRNAs, lncRNAs generally comprise non-protein-coding RNAs that consist of more than 200 nucleotides [13]. Recent reports have indicated that lncRNAs can serve as prognostic markers in various cancer types. Examples include HOTAIR in colorectal cancer and MALAT1 in non-small cell lung cancer [14-15]. HOTAIR and MALAT1 have also been demonstrated to be involved in breast and colorectal cancer metastasis [16-17]. Our previous studies showed that HOTAIR is overexpressed in high-grade glioma patients, and its upregulation is predictive of poor prognosis. In addition, gene set enrichment analysis has indicated that HOTAIR expression is involved in cell cycle progression [18]. However, the precise mechanism underlying how HOTAIR regulates cell cycle progression of glioma cells remains largely unknown. A study by Chang et al. demonstrated that HOTAIR serves as modular scaffold for two histone modification complexes. The 5′ and 3′ domains of HOTAIR bind to the PRC2 and LSD1 complexes, respectively [19]. The HOTAIR-mediated mechanism that regulates cell cycle progression via these two complexes in glioma cells remains unknown.

In this study, we aim to clarify the mechanism underlying HOTAIR-mediated regulation of cell cycle progression in glioma cells, as well as the function of PRC2 and LSD1 in this process. We first found that EZH2 inhibition elicits effects that are consistent with those elicited by HOTAIR-targeted siRNA. We also found that the expression of the 5′ domain of HOTAIR partially rescues the cell cycle arrest induced by HOTAIR knock-down in glioma cells. Thus, we demonstrate that in glioma cells, HOTAIR promotes cell cycle progression in an EZH2-dependent manner. These observations could further our understanding on the regulatory system of cell cycle progression in glioma cells.

## RESULTS

### EZH2 inhibitor blocks GBM cell cycle progression

**Figure 1:**
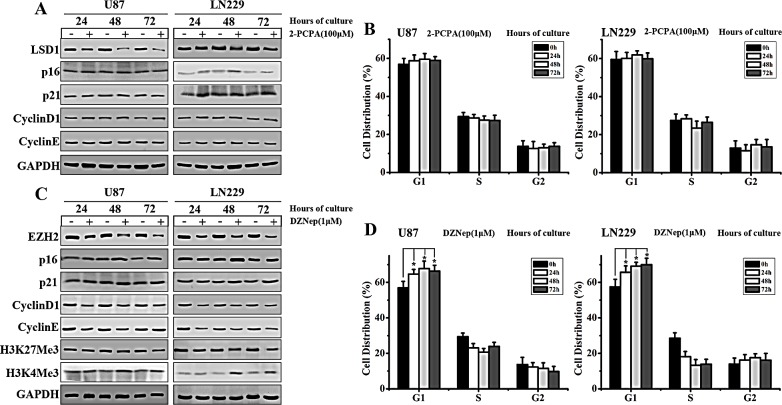
EZH2 inhibition blocks cell cycle progression in GBM cells (A) Western blot analysis of U87 and LN229 cells shows the expression of LSD1, p16, p21, Cyclin D1 and Cyclin E after treatment with 2-PCPA (100 μM) at 24, 48 and 72 h, with GAPDH serving as a loading control. (B) Flow cytometry was performed to examine the G1/S arrest effect in U87 and LN229 cells after treatment with 2-PCPA (100 μM) for 24, 48 and 72 h. (C) Western blot analysis shows the expression of EZH2, p16, p21, Cyclin D1, Cyclin E, H3K27Me3 and H3K4Me3 in U87 and LN229 cells treated with DZNep (1 μM) at 24, 48 and 72 h, with GAPDH as a loading control. (D) Flow cytometry was performed to examine the G1/S arrest effect in U87 and LN229 after treatment with DZNep (1 μM) for 24, 48 and 72 h. The results presented represent mean values ± SD of 3 independent experiments, which were each performed in triplicate. Student's paired t tests were used to calculate P values, where P < 0.05 was considered to be statistically significant.

Previous studies have shown that HOTAIR might serve as scaffold for LSD1 and PRC2 complexes [20-22]. We sought to determine which complex is more crucial for HOTAIR-regulated GBM cell cycle progression. To this end, we used small molecule inhibitors of both LSD1 and EZH2 (main component of PRC2 complex) in our subsequent studies. Consistent with other studies, we used 2-PCPA (LSD1 inhibitor) and DZNep (EZH2 inhibitor) at concentrations of 100 and 1 μM, respectively [23-24]. The GBM cell lines U87 and LN229 were treated with 2-PCPA or DZNep for 24, 48 and 72 h. Western blot analysis revealed that LSD1 expression decreased after 2-PCPA treatment. However, expression of the cyclin-dependent kinase inhibitors p21 and p16 were only slightly changed. In addition, the G1/S-specific proteins Cyclin D1 and Cyclin E expression did not significantly change (Figure 1A). In contrast, in DZNep-treated GBM cells, p16 and p21 were both significantly upregulated at these time points, particularly at 48 h. On the other hand, Cyclin D1 and Cyclin E expression levels were dramatically reduced in cells treated for 48 h (Figure 1C). As EZH2 was previously demonstrated to catalyze H3K27 methylation [25], we also examined the expression of H2K27Me3. The results showed that after DZNep treatment, H2K27Me3 levels decreased, whereas H2K4Me3 levels remained unchanged (Figure 1C). In addition, altered cell cycle distribution at G1 phase was detected in DZNep-treated but not in 2-PCPA-treated, U87 and LN229 cells (Figure 1 B and D). Thus we confirmed that EZH2 but not LSD1 was involved in the cell cycle progression in GBM cells. It was evidenced by the observation that cell cycle progression in GBM cells was significantly blocked after DZNep treatment for 48 h.

### DZNep elicits similar cell cycle effects as si-HOTAIR in GBM cells

**Figure 2:**
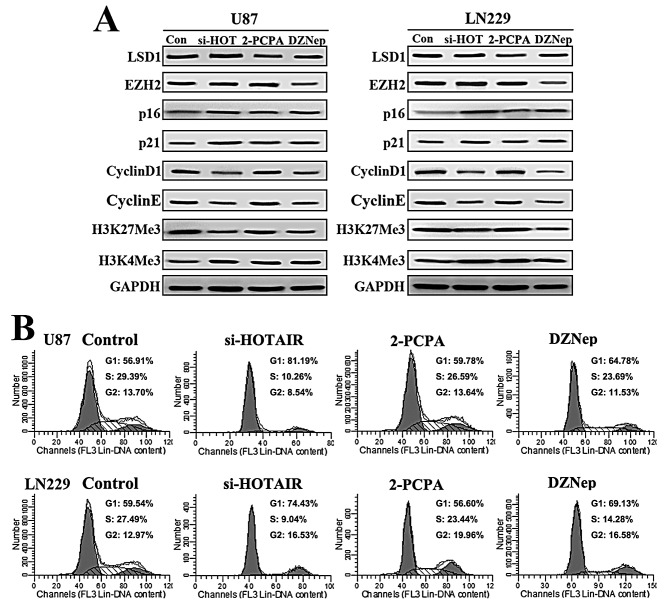
DZNep elicits similar cell cycle effects as si-HOTAIR in GBM cells (A) Western blot analysis was used to examine the expression of LSD1, EZH2, p16, p21, Cyclin D1, Cyclin E, H3K27Me3 and H3K4Me3 in U87 and LN229 cells treated with si-HOTAIR, 2-PCPA (100 μ M) and DZNep (1 μM) for 48 h. GAPDH was used as a loading control. (B) Flow cytometry was performed to examine the G1/S arrest in U87 and LN229 after treatment with si-HOTAIR, 2-PCPA (100μM) and DZNep (1 μM) for 48 h.

Next, we compared the cell cycle effects of HOTAIR siRNA, 2-PCPA and DZNep in GBM cells. U87 and LN229 GBM cells were treated with si-HOTAIR, 2-PCPA (100 μM) and DZNep (1 μM). Western blot analysis revealed that both p16 and p21 protein levels increased after si-HOTAIR and DZNep treatment compared with 2-PCPA. In addition, decreased Cyclin D1 and Cyclin E expression were detected after si-HOTAIR and DZNep treatment (Figure 2A). In addition, we detected that si-HOTAIR inhibited H3K27Me3 to a similar extent as DZNep (Figure 2A). Next, flow cytometry was performed to examine cell cycle distribution. Our results revealed that DZNep elicited a similar G1/S cell cycle arrest as si-HOTAIR (Figure 2B). These data indicate that DZNep treatment elicits similar cell cycle effects as si-HOTAIR in GBM cells.

### HOTAIR inhibition suppresses tumor growth in a xenograft model predominantly via its 5′ domain

**Figure 3:**
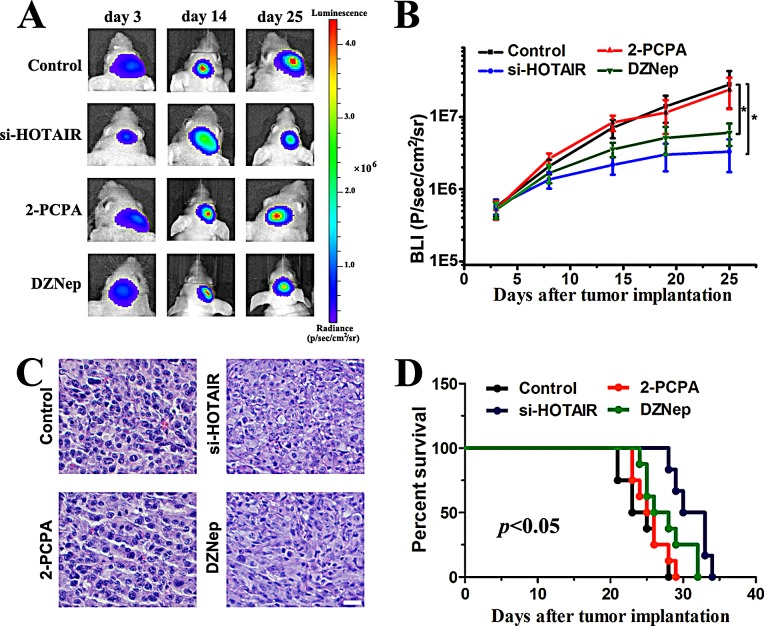
Si-HOTAIR and DZNep treatment prolongs survival in an intracranial glioma murine xenograft model (A) U87 cells pretreated with a lentivirus containing luciferase were implanted in the right forebrain of nude mice, and tumor formation was assessed by bioluminescence imaging. Changes in bioluminescent signal were examined at day 3, 14 and 21 after tumor implantation. (B) The animals were intraperitoneally injected with 2-PCPA, DZNep or PBS (control) in each group, and bioluminescence was monitored to assess the tumor growth at day 3, 8, 14, 19 and 25. *P < 0.05. (C) At day 25, one mouse was sacrificed in each group, and HE staining was used to examine morphological changes. (D) Overall survival was determined by Kaplan-Meier analysis, and log-rank test was used to assess the statistical signiﬁcance of the differences.

To evaluate the effects of si-HOTAIR, 2-PCPA and DZNep on tumor growth *in vivo*, we established intracranial xenograft tumors in nude mice. U87 cells were pretreated with a lentivirus containing a luciferase reporter. All of the mice generated tumors except for two mice within the si-HOTAIR treated group. Compared with 2-PCPA, both si-HOTAIR and DZNep treatments significantly decreased tumor burden (Figure 3A and B). At day 25, one mouse was sacrificed in each group. HE staining revealed apoptotic morphological changes in si-HOTAIR- and DZNep-treated groups (Figure 3C). To analyze the survival times of the treatment groups, we then generated Kaplan-Meier survival curves, which demonstrated that si-HOTAIR and DZNep significantly prolonged survival (Figure 3D). These data showed that DZNep elicited similar effects as si-HOTAIR in regulating tumor growth in GBM xenograft model. Together with the findings in Figure 2, we propose that HOTAIR might modulate cell cycle progression in an EZH2-dependent manner in GBM cells.

### Expression of the 5′ domain of HOTAIR rescues the cell cycle progression in HOTAIR knock-down GBM cells

**Figure 4:**
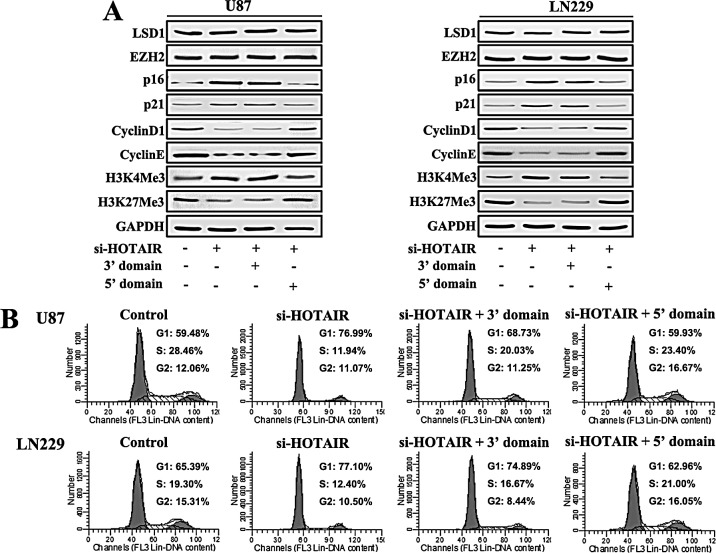
Expression of the 5′ domain of HOTAIR rescues the cell cycle progression of HOTAIR knock-down GBM cells U87 and LN229 GBM cells were infected with si-HOTAIR, and simultaneously transfected with either the 3′ or 5′ domains of HOTAIR. After 48 h of treatment, (A) Western blot analysis was performed on the expression of LSD1, EZH2, p16, p21, Cyclin D1, Cyclin E, H3K4Me3 and H3K27Me3; GAPDH was used as a loading control. (B) Flow cytometry was performed to examine the G1/S arrest effect.

To further confirm whether the regulation of cell cycle progression in GBM cells by HOTAIR is EZH2-dependent, we infected U87 and LN229 cells with si-HOTAIR lentivirus. A plasmid containing HOTAIR 3′ and 5′ domains were transfected at the same time. At 48 h after treatment, protein was prepared for western blot analysis, which revealed that si-HOTAIR blocked GBM cell cycle progress, as shown in Figure 2. After the expression of the HOTAIR 5′ domain, the expression of p21, p16, Cyclin D1 and Cyclin E were almost restored to basal levels. However, the expression of the HOTAIR 3′ domain did not change the expression of these proteins (Figure 4A). Next, flow cytometry was performed to examine cell cycle distribution. Our results showed that the expression of the HOTAIR 5′ domain in si-HOTAIR-transfected cells rescued the G1/S cell cycle arrest (Figure 4B).

### Putative HOTAIR target genes are enriched for cell cycle regulatory function

**Figure 5:**
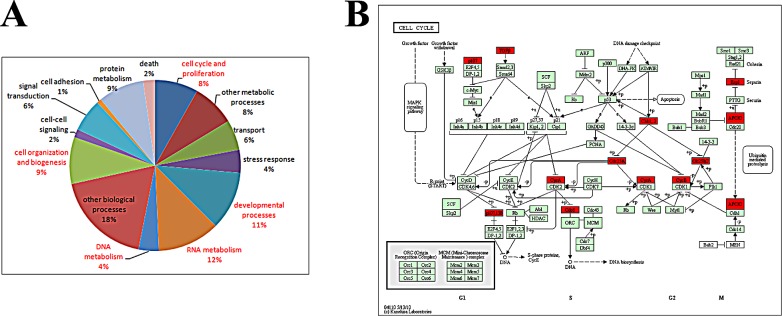
HOTAIR target genes are enriched for cell cycle regulatory genes (A) Gene ontology results revealed that 8% of the target genes are involved in cell cycle and proliferation. The captions of GO terms in red indicate P value <0.05. (B) KEGG pathway analysis indicates that 10 of the target genes (enrichment P value 0.000155) represent key elements in cell cycle progression.

To identify putative HOTAIR target genes, we performed microarray analysis of 220 Chinese glioma samples. Gene ontology (GO) and KEGG analyses were used to annotate these target genes. GO analysis revealed that HOTAIR target genes are involved in cell cycle- and proliferation-related processes (with a P value of 1.86E-07) (Figure 5A and Table 1). KEGG analysis revealed that 10 genes were enriched in cell cycle-related pathways (enrichment P value of 0.000155) (Figure 5B). Taken together, all these data demonstrate that si-HOTAIR inhibits GBM cell cycle progress in an EZH2-dependent manner.

**Table 1:** P values of the Gene Ontology analysis results (genes with P < 0.05 are shown in red)

term	count	P-value
cell cycle and proliferation	47	1.86E-07
other metabolic processes	47	0.593762
transport	33	0.929507
stress response	25	0.117135
developmental processes	64	0.001425
RNA metabolism	68	0.000403
DNA metabolism	22	2.21E-06
other biological processes	106	0.88144
cell organization and biogenesis	51	0.00072
cell-cell signaling	9	0.120696
signal transduction	36	0.999991
cell adhesion	5	0.982058
protein metabolism	50	0.313037
death	13	0.663569

## DISCUSSION

HOTAIR has been extensively demonstrated to correlate with poor prognosis for breast, pancreatic and colon cancer patients [14,16,26]. Furthermore, HOTAIR has been reported to regulate cancer cell cycle progression. However, whether HOTAIR is also involved in cell cycle regulation in glioma cells remains undetermined. This study identifies HOTAIR as a cell cycle-regulating lncRNA that is essential for glioma cell proliferation, indicating that HOTAIR might be a critical player in cell cycle progression in glioma cells.

Previous studies of HOTAIR have demonstrated that it can serve as a clinical prognostic marker. However, the precise regulatory mechanisms remain largely unknown. A study by Chang et al. previously showed that HOTAIR binds to PRC2 and LSD1 complexes via its 5′ and 3′ domains, respectively [19]. Gene set enrichment analysis based on cDNA microarray data from 32 colorectal cancer specimens showed that HOTAIR expression was significantly correlated with genome-wide retargeting of PRC2 genes [14]. Another microarray study of pancreatic cancer showed that GDF15 was regulated by both HOTAIR and PRC2. In contrast, interferon-related genes were not found to be affected by EZH2 knock-down but by HOTAIR-mediated suppression. These results indicated that HOTAIR-mediated gene repression in pancreatic cancer is both PRC2-dependent and PRC2-independent [26]. However, these studies focused on the HOTAIR-PRC2 axis, while the HOTAIR-LSD1 axis remained unclear. In this study, we examined both HOTAIR-PRC2 and HOTAIR-LSD1 cell cycle regulatory functions in GBM. Our results suggest that in GBM cells, HOTAIR regulates cell cycle progression in an EZH2-dependent manner.

EZH2 is the catalytic subunit of PRC2, which functions as methyltransferase by adding three methyl groups to lysine 27 of histone 3, a modification leading to chromatin condensation [27]. Previous studies have shown that EZH2 is overexpressed in glioma stem-like cells and adult glioblastoma patient samples [28-29]. The inhibition of EZH2 has been shown to induce cell cycle arrest at G0/G1 phase in U87 human glioma cells [30]. In addition, EZH2 phosphorylation activates STAT3 signaling via STAT3 methylation and promotes the tumorigenicity of glioblastoma stem-like cells [31]. However, the function of LSD1 in human glioma remains largely unknown. A study by Singh et al. previously showed that LSD1 inhibition sensitized glioblastoma cells to histone deacetylase inhibitors [32]. In this study, we introduced small molecule inhibitor for both EZH2 and LSD1. GBM cells were treated with si-HOTAIR, an EZH2 inhibitor (DZNep) and a LSD1 inhibitor (2-PCPA). Our results demonstrate that DZNep elicits similar effects as those of si-HOTAIR in terms of glioma cell cycle distribution, whereas 2-PCPA only slightly altered cell cycle distribution. However, studies have shown that the proliferation of neuroblastoma and breast cancer cells was inhibited after treated with LSD1 inhibitor (TCP or TCP analogues) at concentrations 20-30-fold higher than enzymatic IC50 [33]. In HOTAIR knock-down glioma cells, unlike expression of the 5′ domain, the expression of the HOTAIR 3′ domain (LSD1 complex-binding domain) did not rescue the cell cycle arrest. These results demonstrate that in GBM cells, HOTAIR regulates cell cycle progression predominantly via the HOTAIR 5′ domain-PRC2 axis, which is EZH2-dependent.

In this study, we confirmed HOTAIR 5′domain-PRC2 as a new regulatory axis that modulates cell cycle progression in GBM cells. A study by Li et al. showed that HOTAIR is overexpressed in laryngeal squamous cell carcinoma and regulates PTEN methylation [34]. A study by Lu et al. also showed that HOTAIR is involved in gene methylation in breast cancer [35]. However, these studies did not show why HOTAIR is relevant to methylation. As EZH2 is a methyltransferase, based on our findings in this study, we speculate that HOTAIR might also be related to gene methylation via the HOTAIR 5′ domain-EZH2 axis.

Our *in vivo* study also showed that HOTAIR inhibition was therapeutically beneficial. The inhibition of HOTAIR slowed tumor growth and prolonged survival in a xenograft model. Our study suggests another lncRNA-based gene therapy approach for glioma patients. Because HOTAIR regulates cell cycle progression in GBM cells via its 5′ domain, further examination and determination of the structure of HOTAIR, followed by molecular docking-based virtual high-throughput screening techniques, might facilitate the discovery of small molecule inhibitors for HOTAIR.

Our present work uncovers a novel HOTAIR-mediated mechanism of cell cycle regulation in GBM cells and provides a strong rationale for the further development of therapeutic strategies directly or indirectly targeting HOTAIR in GBM, by applying either DZNep or small molecule inhibitors for HOTAIR based on *in silico* 3D structural predictions.

## Materials and methods

### Cell culture and drug treatment

Human glioma cells (U87 and LN229) were obtained from ATCC (the American Type Culture Collection, Manassas, VA, USA) and were cultured in Dulbecco's modified Eagle medium (DMEM) supplemented with 10% heat-inactivated fetal bovine serum (FBS, Hyclone). The cells were maintained in a humidified atmosphere at 10% CO_2_ atmosphere at 37 °C. The LSD1 inhibitor 2-PCPA (Sigma-Aldrich, USA) was dissolved in H_2_O. The EZH2 inhibitor DZNep (Cayman, Michigan, USA) was dissolved in DMSO. The cells were treated with 100 μM 2-PCPA or 1 μM DZNep for 24, 48 or 72 h.

### Clinical samples and bioinformatics

Two hundred and twenty glioma samples were collected from the Chinese Glioma Genome Atlas (CGGA, http://www.cgcg.org.cn/). As we previously described [36], there were 58 astrocytomas, 17 oligodendrogliomas, 22 oligoastrocytomas, 8 anaplastic astrocytomas, 11 anaplastic oligodendrogliomas, 15 anaplastic oligoastrocytomas, 4 secondary, and 85 primary GBMs. RNA was extracted from all of these tumor samples, and the Agilent Whole Human Genome Array was used for microarray analysis following the manufacturer's protocols. The GSEABase package from R (http://www.r-project.org/) statistical platform was used for Gene Ontology (GO) analysis. GO is to perform enrichment analysis on gene sets. GSEABase package provides classes and methods to support Gene Set Enrichment Analysis (GSEA). GSEA is a computational method that could be used to determine whether there is statistically significant difference on a defined set of genes between two biological states. In the set of genes that are up-regulated by HOTAIR, an enrichment analysis will find which GO terms are over-represented by using annotations for that gene set. We also examined these genes in Matlab and mapped them to Kyoto Encyclopedia of Genes and Genomes (KEGG; http://www.genome.jp/kegg/) pathway database to analyze the correlation [37].

### Lentiviral infection and gene transfection

Lentivirus containing HOTAIR siRNA segments (HOTAIR siRNA sequence is 5′-GAACGGGAGUACAGAGAGAUU-3′) was obtained from Genepharma (Shanghai, China). U87 and LN229 cells were infected with the viral suspension. HOTAIR 3′ domain (nucleotides 1 to 300 of HOTAIR) and 5′ domain (nucleotides 1500 to 2146 of HOTAIR) were inserted into pcDNA3.1 (+) plasmid. pcDNA3.1 (+)-3′ domain and pcDNA3.1 (+)-5′ domain plasmids were transfected using Lipofectamine 2000 (Invitrogen, Carlsbad, CA) following the manufacturer's protocols.

### Western blot

Protein lysates were prepared as previously described [38]. The protein samples were resolved by SDS-PAGE and transferred onto PVDF membranes (Roche, Basel, Switzerland). The membranes were then incubated with the following antibodies: anti-LSD1 (Cell Signaling Technology), anti-EZH2 (Cell Signaling Technology), anti-p16 (Santa Cruz), anti-p21 (Santa Cruz), anti-Cyclin D1 (Santa Cruz), anti-Cyclin E (Santa Cruz), anti-H3K27Me3 (Millipore), and anti-H3K4Me3 (Millipore). Antibody-labeled protein bands on the PVDF membranes were detected using a G:BOX F3 (Syngene, Cambridge, UK).

### Cell cycle distribution

U87 and LN229 cells (1×10^5^ cells) were plated in 60-mm culture plates, and the cells were treated as previously described. After 2 days, the cells were trypsinized, fixed in 70% ethanol, washed once with PBS, and then labeled with propidium iodide (Sigma-Aldrich) in the presence of RNase A (Sigma-Aldrich) for 30 min in the dark (50 g/mL). Samples were run on a FACScan flow cytometer (Becton-Dickinson, FL, NJ, USA), and the percentages of cells within each phase of the cell cycle were analyzed using Cell Quest software.

### Intracranial mice model

All protocols involving animals were performed in accordance with an approved Institutional Animal Care and Use Committee protocol. Intracranial transplantation of GBM cells to establish GBM xenografts was performed as described previously [39-40]. Fifty thousand U87 cells (pretreated with either lentivirus containing with or without si-HOTAIR segments) were injected under the guidance of a stereotactic instrument. Three days after inoculation of the U87 cells (day 0), the treatments were initiated as follows: 2-PCPA (2 mg/kg) was injected intraperitoneally daily for 3 weeks, and DZNep was injected intraperitoneally (2 mg/kg) twice per week for 3 weeks. Bioluminescence imaging was used to detect intracranial tumor growth as previously described [40]. Identical circular regions of interest were drawn around the entire head of each animal to quantify bioluminescence. The Living Images software package (Caliper Life Sciences) was used to determine the integrated flux of photons (photons per second) within each region of interest. The data were normalized to the bioluminescence at the initiation of treatment for each animal. The error bars shown in the figures indicate the standard deviation (SD). A Kaplan-Meier survival curve is shown.

### HE staining

The xenograft samples were collected at day 25 after tumor implantation, subjected to HE staining. For staining, 5-μm sections were cut, dehydrated, deparaffinized, and rehydrated. HE staining was performed according to the standard protocols. All images were captured via microscopy (Olympus).
